# PCSK9 in T-cell function and the immune response

**DOI:** 10.1186/s40364-024-00712-8

**Published:** 2024-12-31

**Authors:** Yuying Wang, Xiaosheng Fang, Jiarui Liu, Xiao Lv, Kang Lu, Yingxue Lu, Yujie Jiang

**Affiliations:** 1 Shandong Provincial Hospital Affiliated to Shandong First Medical University, Shandong, 250021 China; 2 Department of Hematology, Shandong Provincial Hospital Affiliated to Shandong First Medical University, Shandong, 250021 China; 3 Department of Nephrology, Shandong Second Provincial General Hospital, Jinan , Shandong, 250021 China

**Keywords:** PCSK9, T cells, Immune response, Signaling pathway, PCSK9 inhibitors

## Abstract

Proprotein convertase subtilisin/kexin type 9 (PCSK9) was first reported in 2003 and confirmed to be strongly associated with familial hypercholesterolemia. Small-molecule inhibitors targeting PCSK9 provide an effective and safe method for managing hypercholesterolemia and reducing the cardiovascular risk. In recent years, increasing evidence has indicated other important roles for PCSK9 in inflammation, tumors, and even immune regulation. PCSK9 might be an attractive regulator of T-cell activation and expansion. It might mediate inflammation and regulate other types of immune cells. In this review, we summarize the current advances in the field of PCSK9 and provide a narrative of the biological processes associated with PCSK9. The relationships between PCSK9 and different T cells were investigated in depth. Finally, the signaling pathways associated with PCSK9 and the immune response are also summarized in this review.

## Introduction

Proprotein convertase subtilisin/kexin type 9 (PCSK9) is a serine protease that was originally named neuroapoptosis-regulated converting enzyme-1 (NARC-1). It was shown to be a member of the proprotein convertase (PC) family, which cleaves a variety of substrates, such as growth factors, transcription factors, enzymes, hormones, and receptors. PCSK9 is the most specific of its family members due to its distinctive self-activation that can make it its exclusive substrate [1]. PCSK9 is mainly expressed and secreted by hepatocytes. In addition, it can be expressed in the intestinal epithelium, pancreatic cells, nerve cells, renal interstitial cells, and embryonic cells. The main function of PCSK9 is to regulate the expression of low-density lipoprotein receptor (LDLR) [2, 3]. Small-molecule inhibitors targeting PCSK9 provide an effective and safe method for managing hypercholesterolemia and reducing the cardiovascular risk.

In recent years, increasing evidence has indicated other important roles for PCSK9 in inflammation, tumors, and even immune regulation. Oxidized low-density lipoprotein (OxLDL) activation is associated with the immune effects of PCSK9 [4]. The immunomodulatory effect of PCSK9 may also depend on low-density lipoprotein (LDL) heterogeneity [5]. Previous studies confirmed that PCSK9 could regulate the proliferation, differentiation, and apoptosis of multiple subtypes of T cells, including CD4 + /CD8 + T cells, cytotoxic T lymphocytes (CTLs), and helper T cells (Th)/regulatory T cells (Tregs). Therefore, PCSK9 might play an important role and might be an attractive target for investigating the complex immune response. However, most of the literature has focused mainly on lipid metabolism, and only a few articles have mentioned the associations among tumorigenesis, autoimmune diseases, and infectious diseases.

Here, we summarize the current research progress on PCSK9, and the relationships between PCSK9 and T-cell activation, as well as signaling pathways, are also discussed.

## Biological processes associated with PCSK9

### PCSK9 and lipid metabolism

PCSK9 was first identified by Seidah NG et al. in 2003 [6] and was confirmed to play a crucial role in the regulation of lipid metabolism. PCSK9 has received increased attention from cardiovascular specialists because of its specific mechanisms of action in lipid metabolism.

**Fig. 1:**
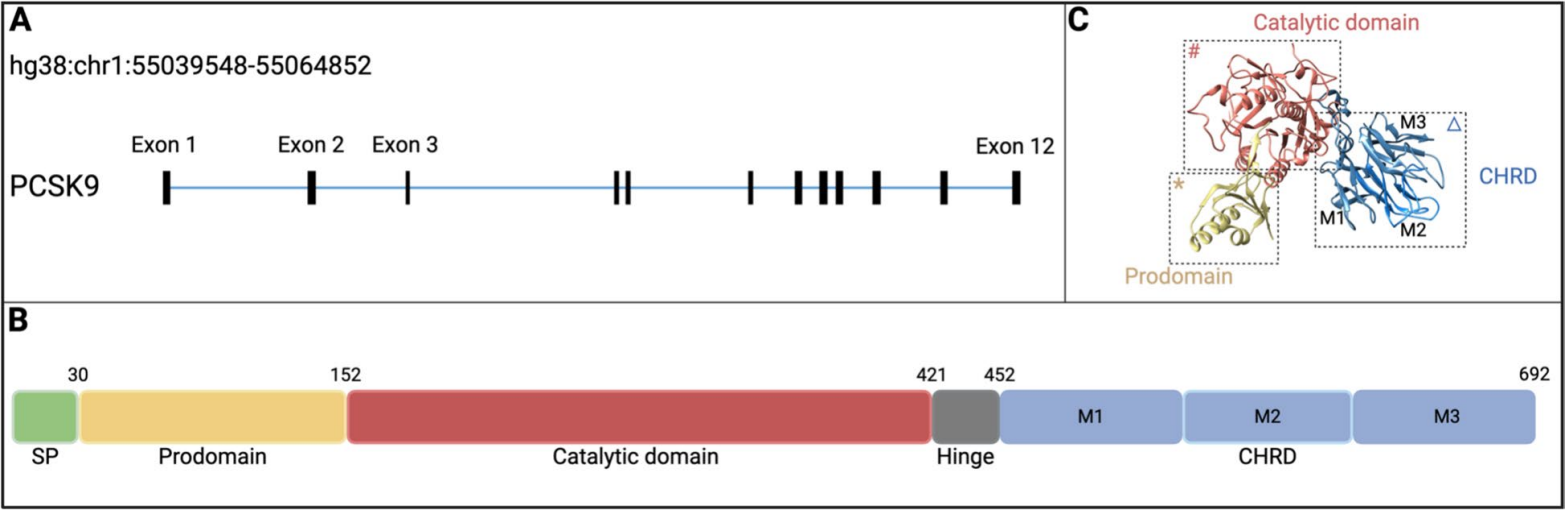
The gene and protein structures of PCSK9. **A** Schematic representation of PCSK9 gene structure. The PCSK9 gene is located on chromosome 1 (hg38) within the genomic region chr1:55039548–55064852. It consists of 12 exons (black) and 11 introns (blue). The coding sequence (CDS) of the gene spans 2079 base pairs (bp) and encodes a protein comprising 692 amino acids. **B** Structure domains of human PCSK9. PCSK9 contains a signal peptide (SP; aa 1–30), a prodomain (aa 31–152), a catalytic structural domain (aa 153–421) with a hinge (aa 422–452), and a C-terminal Cys/His-rich-domain (CHRD; aa 453–692) that is composed of three modules M1 (aa 453–529), M2 (aa 530–603), and M3 (aa 604–692). **C** The crystal structure of PCSK9. The prodomain is in yellow, the catalytic domain is in red, and the CHRD is in blue (Figure from PDB ID: 2PMW). ^*^ Prodomain: most of the GOF and LOF mutations of PCSK9 are occurred in this region. ^**#**^ Catalytic region: many small molecule drugs (e.g. mAbs) are designed to bind to the LDLR at this location, thereby affecting LDL degradation. ^**Δ**^ CHRD: most PCSK9 mutations occur in the M1 and M3 modules, while fewer PCSK9 genetic variants are observed in the hinge region and M2 module, but with greater structural flexibility

The PCSK9 gene structure and the three-dimensional (3D) structures of PCSK9 are shown in Fig. 1. The cDNA of PCSK9 was initially found in projects studying apoptosis in cerebellar neurons and secreted proteins, and was eventually identified in human, mouse, and rat libraries [6]. The 22-kb human PCSK9 gene is located on chromosome 1p32 and consists of 12 exons and 11 introns. Its mRNA is 3,710 base pairs (bp) in length and its coding sequence (CDS) is 2,079 bp in length, and encodes a protein of 692 amino acids (aa) [7, 8] (Fig. 1A). PCSK9 comprises a signal peptide (SP), an N-terminal domain (prodomain), a catalytic structural domain with a hinge, and a C-terminal Cys/His-rich-domain (CHRD) that can be further divided into three repeat modules, M1, M2, and M3 [7, 9, 10] (Fig. 1B). In the endoplasmic reticulum (ER), the prodomain is associated with PCSK9 maturation and mature PCSK9 maintains its association with the prodomain after being secreted [11, 12]. The prodomain plays a key role in regulating PCSK9 activity, secretion, gain of function (GOF) and loss of function (LOF) mutations, and interaction with LDLR [6, 13]. The catalytic domain is important in designing new agents targeting PCSK9, e.g., PCSK9 monoclonal antibodies (mAbs) can block the interaction between the catalytic domain of PCSK9 and LDLR epidermal growth factor-like repeat A (EGF-A) structural domain to negate PCSK9’s activity against LDLR recycling [10]. The modules M1, M2, and M3 bear a structural resemblance to resistin, a secreted small protein to modulate mammalian glucose metabolism. The majority of PCSK9 variations found within the CHRD occur within the M1 and M3 modules, whereas fewer PCSK9 genetic variations but greater structural flexibility is observed in the hinge region and M2 module [10, 14–16] (Fig. 1C).

PCSK9 regulates cholesterol metabolism by binding to LDLR through both extracellular and intracellular pathways. The extracellular pathway is generally utilized by hepatic, pancreatic, and intestinal epithelial cells. After release from the Golgi apparatus, PCSK9 can directly bind to LDLR expressed on the cell surface, then fuse with lysosomes, and finally enter the degradation process. Thus, it prevents the recirculation of LDLR to the cell surface and blocks the main pathway of low-density lipoprotein cholesterol (LDL-C) export from the circulation, resulting in an increase in LDL-C levels in the blood [17]. However, in the intracellular pathway, secreted/plasma PCSK9 binds to the EGF-A domain of LDLR via its catalytic subunit, which better transports LDLR to the lysosome and mediates its degradation [3, 18]. Another study indicated that a third pathway critical for PCSK9-induced LDLR degradation was mediated by an adenylate cyclase-associated protein 1 (CAP1)-dependent protein with a globular C-terminus structurally similar to the C-terminal CHRD of PCSK9. CAP1 can bind to the CHRD and promote degradation of the PCSK9-LDLR complex in lysosomes. The authors identified CAP1 as a new binding partner of PCSK9 and a key mediator of the caveolae-dependent endocytosis and lysosomal degradation of LDLR [19].

**Fig. 2:**
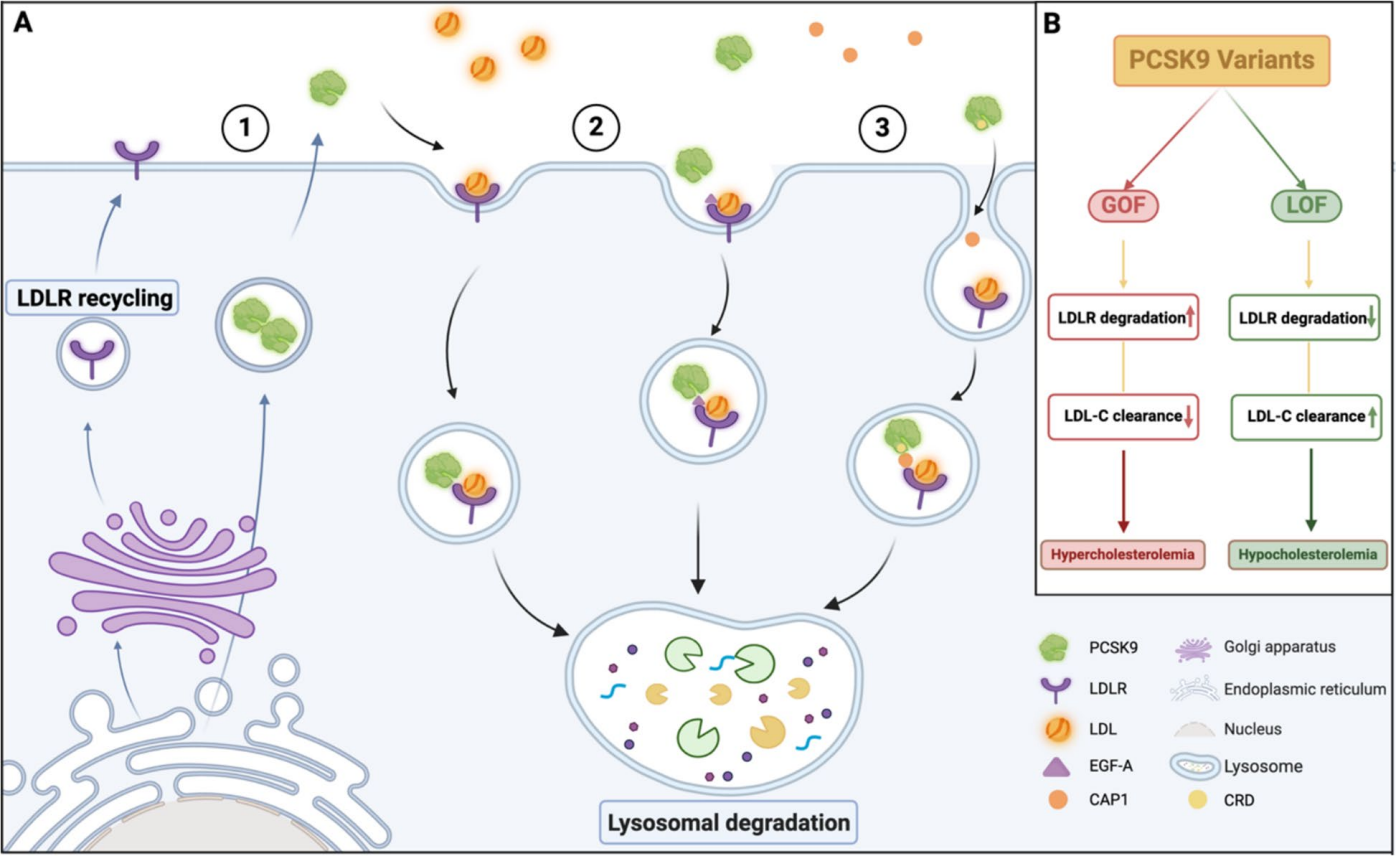
The mechanism and effect of PCSK9 on regulating cholesterol metabolism (created in https://BioRender.com). PCSK9 binds to LDLR and regulates cholesterol metabolism through extracellular and intracellular pathways. ① After PCSK9 is released from the Golgi apparatus and binds directly to LDLR expressed on the cell surface, it fuses with lysosomes and enters the degradation pathway. ② PCSK9 binds to the EGF-A domain of LDLR through its catalytic subunit, transports LDLR to the lysosome, and mediates its degradation. ③ CAP1 binds to CHRD and promotes degradation of the PCSK9-LDLR complex in lysosomes. In addition, PCSK9 GOF and LOF mutations lead to the upregulation and inhibition of LDLR degradation, respectively, promoting hypercholesterolemia and hypocholesterolemia, respectively

Previous studies have shown that the overexpression or underexpression of PCSK9 has crucial effects not only on circulating PCSK9 and LDL-C levels but also on cardiovascular diseases such as familial hypercholesterolemia (FH), metabolic syndrome, and coronary atherosclerotic heart disease (CHD) [20]. PCSK9 itself can be mutated, including GOF and LOF. GOF mutations result in the enhancement of the ability of PCSK9 to upregulate LDL-C, leading to hypercholesterolemia. LOF mutations reduce LDLR degradation and enhance plasma clearance of LDL-C, thereby reducing LDL-C levels and leading to hypocholesterolemia [21]. Seidah NG et al. reported 37 missense mutations in the N-terminal acidic prodomain including both GOF and LOF variants of PCSK9 [22]. The mechanism and effect of PCSK9 regulating cholesterol metabolism are shown in Fig. 2.

### PCSK9 inhibitor development

Due to its distinct roles in lipid metabolism, PCSK9 is an important pharmacological target for lowering LDL-C levels and stabilizing plaques. New agents targeting PCSK9 have significantly improved the symptoms and prognosis of patients with major cardiovascular diseases. Many clinical trials of monoclonal antibodies against PCSK9, including evolocumab and alirocumab, have been reported [23–25].

Evolocumab (AMG 145) and alirocumab (SAR236553/REGN727) are fully human immunoglobulin G2 and G1 mAb, respectively. The molecular profile of evolocumab consists of two disulfide-bonded human heavy chains and each of them is covalently linked to a kappa light chain [26, 27]. They have similar molecular characteristics and are not metabolized in the liver or kidney, do not penetrate the central nervous system, and do not affect cardiac function. There are no drug-drug interactions between these two drugs because they do not interact with cytochrome P450 enzymes or transporter proteins [27–29]. Sabatine et al. reported that evolocumab decreased LDL-C levels by 61%, and the incidence of cardiovascular events decreased from 2.18% in the control group to 0.95% after 1 year of treatment (*p* = 0.003) [30]. Compared with the placebo, evolocumab treatment significantly reduced the risk of cardiovascular death, myocardial infarction, hospitalization for unstable angina, and coronary revascularization (*p* < 0.001) [31]. According to a post hoc analysis of one trial, the rate of major adverse cardiovascular events (coronary heart disease-related death, myocardial infarction, unstable angina requiring hospitalization, or ischemic stroke) was lower in the alirocumab group than in the placebo group (*p* = 0.02) [32].

Inclisiran, a double-stranded small interfering RNA (siRNA), inhibits the synthesis of PCSK9 through gene silencing. In the cytoplasm of hepatocytes, the antisense strand of inclisiran binds to PCSK9 mRNA, ultimately resulting in its breakdown [33]. The ORION series of trials investigated the effects of Inclisiran on lowering LDL-C levels and cardiovascular outcomes. Approved drugs for the treatment of certain types of hypercholesterolemia include evolocumab, alirocumab, and inclisiran. Other therapies are in preclinical or clinical trial stages. These drugs include additional monoclonal antibodies, small molecule inhibitors, antisense oligonucleotides, binding peptides (adnectins), vaccines, and gene editing therapies. The mechanisms, details of the associated trials, and major side effects of different PCSK9 inhibitors are listed in Table 1. Among them, vaccines and gene editing therapies hold particular promise and may potentially achieve lifelong low expression with a single treatment [34].

**Table 1:** PCSK9 inhibitors in clinical/preclinical trials

	**Mechanism**	**Agent/compound (Ref.)**	**Trials**	**Patient population/subject**	**Side effects**
Monoclonal antibodies	Promote LDL-C clearance by binding to the catalytic domain of PCSK9 and preventing its interaction with LDLR on the surface of hepatocytes [32]	Evolocumab [25, 35]	1. DESCARTES 2. FOURIER	1. Patients with hyperlipidemia 2. Patients with ASCVD and high LDL-C on statin treatment	Injection-site reactions, nasopharyngitis, and upper respiratory infections
		Alirocumab [36, 37]	1. ODYSSEY OUTCOMES 2. PACMAN	1. Patients at high risk for CV events with high LDL-C levels 2. Patients with AMI	
		Bococizumab [38, 39]	1. SPIRE-1 and SPIRE-2 2. SPIRE	1. Patients with ASCVD 2. Patients with hyperlipidemia	
		Frovocimab [40]	A study of LY3015014 in participants with high cholesterol	Patients with primary hypercholesterolemia on statin therapy	
		RG7652 [41]	A phase 1, randomized, double-blind, placebo-controlled, ascending-dose study of RG7652	Healthy subjects with normal serum LDL-C levels	
Binding peptides	Designed to bind PCSK9 and block its interaction with LDLR	MK-0616 [42, 43]	1. A single rising-dose Phase 1 clinical trial of MK-0616 2. Study of the efficacy and safety of MK-0616 in adults with hypercholesterolemia	1. Healthy adult participants 2. Patients at risk of or with ASCVD	Need further confirmation
		BMS-962476 [44]	Preclinical trials	Genomic transgenic mice; Cynomolgus monkeys	
Gene silencing	Use siRNA to reduce the production of PCSK9 protein by decreasing PCSK9 mRNA levels	Inclisiran [45–47]	ORION-1	High-risk ASCVD patients with elevated LDL-C levels	Injection-site reactions
			ORION-9	Patients with heterozygous FH	
			ORION-10 and ORION-11	Patients with ASCVD or an ASCVD risk equivalent with elevated LDL-C	
Gene editing	Direct modification of the PCSK9 gene using CRISPR/Cas9 technology [48]	VERVE-101 [49]	Heart-1 clinical trial	Patients with FH and ASCVD	Need further confirmation
Active immunization	Development of vaccines to induce antibody production against PCSK9	AT04A anti-PCSK9 vaccine [50]	Preclinical trials	CETP mice	Need further confirmation
		L-IFPTA vaccine [51]		Hypercholesterolemic and atherosclerotic mice	

*Ref* Reference, *LDL-C* Low-density lipoprotein cholesterol, *LDLR* Low-density lipoprotein receptor, *siRNA* Small interfering RNA, *ASCVD* Atherosclerotic cardiovascular disease, *AMI* Acute myocardial infarction, *FH* Familial hypercholesterolemia, *L-IFPTA* Liposomal Immunogenic Fused PCSK9-Tetanus peptide plus Alum adjuvant, *CETP* Cholesterol ester transfer protein

Landlinger C. and his colleagues reported that PCSK9 vaccination might be applied as a permanent method of silencing PCSK9 with effective and long-lasting therapeutic effects [50]. A study published in *Nature* reported that the vaccination of mice with a virus-like particle (VLP)-peptide vaccine targeting PCSK9 resulted in significantly lower LDL cholesterol levels and increased levels anti-inflammatory factors [52]. Their study showed that this vaccination could induce an obvious improvement in atherosclerotic lesions and a long-lasting therapeutic effect [53]. Similarly, Mahboobnia K. confirmed the effect and safety of long-term specific antibodies in mouse models vaccinated with PCSK9 [54]. With the widespread use of PCSK9 inhibitors, long-term safety studies will continue to ensure that there are no unforeseen long-term side effects. For example, VERVE-101, which was generated by CRISPR/Cas9 technology, could directly modify the PCSK9 gene. However, such approach often faces a series of challenges such as off-target effects and ethical issues.

In summary, due to the crucial role of PCSK9 in lipid metabolism and the developed drug therapy, it holds great promise asavaluable tool for lipid management, prevention, and treatment of related diseases caused by abnormal lipid metabolism. In the future, more researches and refinements are needed before these new approaches could be translated into clinical applications.

### PCSK9 and tumorigenesis

PCSK9 upregulation has been confirmed in various types of solid malignancies. Some biological processes associated with apoptosis, inflammation, and the stress response are important in tumorigenesis. Liu et al. confirmed that the inhibition of PCSK9 could enhance the synergistic antitumor effect of programmed cell death-1 (PD-1) inhibitors (immune checkpoint inhibitors [ICIs]) in colorectal cancer (CRC). They found that the inhibition of PCSK9 enhanced the tumor response to PD-1 inhibitors by promoting massive infiltration of CTLs within the tumor through a mechanism independent of the cholesterol regulatory function of PCSK9 [55]. Another study knocked out the PCSK9 gene in four malignant mouse cancer cell lines (B16F10, 4T1, MC38, and CT26) using CRISPR-Cas9 technology, and tumor growth was significantly attenuated or blocked in mice in a CTL-dependent manner and the efficacy of PD-1 inhibitors was enhanced [48]. In all four models, PCSK9-neutralizing antibodies synergistically inhibited tumor growth with anti-PD-1 therapy [48].

Bhat M et al. analyzed tissue samples from patients with hepatocellular carcinoma (HCC) who underwent partial hepatectomy or liver transplantation and reported decreased PCSK9 expression and increased LDLR expression in HCC. They showed that the metabolism of HCC and its growth potential could be effectively inhibited by treatment with PCSK9 inhibitors [56]. Non-small cell lung cancer (NSCLC) is the most common lung cancer [57]. Gao et al. documented that high expression of PCSK9 in tumor tissues was a deleterious factor in the efficacy of anti-PD-1 immunotherapy in patients with advanced NSCLC. A total of 115 patients with advanced NSCLC receiving anti-PD-1 immunotherapy were included, and PCSK9 expression in baseline NSCLC tissues was detected using immunohistochemistry (IHC). Their results showed that the median progression-free survival (mPFS) in the PCSK9 low-expression group was significantly longer than that in the PCSK9 high-expression group (8.1 *vs.* 3.6 months). The objective response rate (ORR) and disease control rate (DCR) were significantly higher in the PCSK9 low-expression group than in the PCSK9 high-expression group (54.4% *vs.* 34.5% and 94.7% *vs.* 65.5%, respectively) [58]. They found that PCSK9 inhibitors combined with anti-CD137 agonists not only enhanced the recruitment of CD8-positive (CD8 +) T cells, but also depleted Tregs. This may be a novel therapeutic strategy for future research and clinical practice.

Vaccines targeting PCSK9 were also investigated in tumor cells. Momtazi-Borojeni et al. produced specific antibodies against PCSK9 in C57BL/6 mice treated with nanoliposomal PCSK9 vaccines. Two weeks after the final immunization, the mice were subcutaneously inoculated with B16F0 melanoma cells. Their results confirmed that PCSK9 deficiency could reduce the metastasis of melanoma in the livers of mice [59]. Their previous experimental studies demonstrated that L-IFPTA + vaccine-mediated inhibition of PCSK9 moderately suppresses tumor growth and improves lifespan and survival in mice with breast and colon cancers [60, 61]. The mechanisms underlying these findings were related to the fact that the L-IFPTA + vaccine triggered functional anti-PCSK9 antibodies targeting circulating PCSK9, thereby reducing the plasma levels and activity of PCSK9 in mice. Further preclinical and clinical studies are needed for PCSK9 vaccines to ensure the efficacy and safety of therapeutic inhibition of PCSK9 in patients with different types of cancers.

In addition, apoptosis might be involved in the underlying pathogenetic mechanism in the existing studies of PCSK9 and tumorigenesis. Xu et al. reported that high expression of PCSK9 in gastric cancer (GC) tissues was associated with cancer progression and a poor prognosis, and they proposed that PCSK9 might be a potential therapeutic target for GC [62]. The underlying mechanism might be related to the inhibition of tumor cell apoptosis by high expression of PCSK9. Sun et al. demonstrated that PCSK9 has anti-apoptotic effects in mouse liver. They injected B16F1 melanoma cells into PCSK9 KO mice to induce liver metastasis and found that the mice had a low risk of developing liver metastases, which correlated with cholesterol levels. Their findings derived from the reduction in cholesterol levels and the enhancement of tumor necrosis factor α (TNF-α)-mediated apoptosis in the absence of PCSK9, which is detrimental to tumor development [63].

A study to explore the precise role of PCSK9 in lung cancer cell apoptosis confirmed that PCSK9 siRNA had antitumor activity and induces increased apoptosis in A549 cells. The mechanism might be related to the activation of caspase-3, down-regulation of the anti-apoptotic protein survivin and the x-linked apoptosis inhibitory protein [64]. PCSK9 could be used as a therapeutic target for prostate cancer. Gan et al. found that PCSK9 siRNA had a protective effect on ionizing radiation-induced PCa cell damage in prostate cancer by reducing apoptosis [65]. To date, several studies have confirmed that the inhibition of PCSK9 attenuates the progression of breast cancer, glioma, colon tumors, and prostate cancer [66]. Due to the potential mechanisms explored and the important role played by PCSK9 in tumorigenesis, more preclinical and clinical studies are now needed to develop drug therapies.

### PCSK9 and inflammatory processes

Inflammation is a component of many biological processes, including infection, the pathophysiology of atherosclerosis, autoimmune diseases, and tumorigenesis [67]. PCSK9 regulates the concentration of sirtuins, a family of proteins involved in histone deacetylation, and plays a key role in metabolically driven inflammation [68, 69]. PCSK9 plays an important role in both infectious and noninfectious inflammatory diseases. However, the exact mechanism by which PCSK9 is involved in oxidative and inflammatory mechanisms is uncertain [70].

Previous studies have revealed a possible correlation between PCSK9 and the development of infectious diseases, and PCSK9 is expected to be a therapeutic target [71, 72]. One decade ago, sepsis was considered a serious multiorgan dysfunction syndrome caused by infectious factors, and the routine and major treatment strategy for sepsis was antibiotic therapy. In 2014, Walley et al. first proposed that PCSK9 is a critical regulator of the innate nervous system and mediates the whole immune response to infectious shock. Their results showed that reduced PCSK9 function was associated with accelerated pathogen lipid clearance via LDLR, a decreased inflammatory response, and improved septic shock outcomes [73]. Another study proposed the exciting hypothesis that the silencing of PCSK9 expression or inhibitors targeting PCSK9 can both enhance lipid clearance by pathogens through LDLR and shorten the inflammatory process [3]. Interestingly, PCSK9 has been confirmed to be a sensitive and independent predictive risk factor for the prognosis of patients in the early stages of sepsis [74]. Yuan et al. reported that PCSK9 might be an adverse factor for the survival of the immune response during sepsis, suggesting that it may be a potential target for sepsis therapy [75].

The formation of atheromatous plaques is also accompanied by inflammation. PCSK9 inhibitors have been shown to improve the inflammatory status of patients with FH [76]. Recent research confirmed that PCSK9 is associated with inflammation and that it promotes inflammatory responses by interacting with scavenger receptors (SRs) of inflammatory mediators such as lectin-like oxidized LDL receptor-1 (LOX-1) [77]. OxLDL is an antigen that may play a major role in immune activation in individuals with atherosclerosis [78]. Liu P et al. reported that dendritic cells (DCs) and T cells are present in atherosclerotic plaques and might play important roles in different stages of atherosclerosis development [79]. Liu et al. showed that the immune effects of PCSK9 are associated with oxLDL activation and mature DCs and plaque T cells. Human DCs can produce PCSK9 after oxLDL stimulation, suggesting that DCs can be involved in local immune activation and oxLDL-induced atherosclerotic plaques [4, 80]. Another immunological study reported that oxLDL induces PCSK9 in DCs and induces DC maturation. Silencing of PCSK9 inhibited the oxLDL-induced activation of T cells by DCs and promoted an anti-inflammatory phenotype [81].

In addition to its role in infectious sepsis and atherosclerosis, PCSK9 is involved in other noninfectious conditions, such as autoimmune diseases. Lee et al. investigated the role of PCSK9 in the pathogenesis of Graves orbitopathy (GO) and proposed that PCSK9 could serve as a therapeutic target and biomarker for GO. They confirmed that the production of proinflammatory cytokines, oxidative stress, and fibroblast differentiation could be attenuated by the inhibition of PCSK9 [82].

Systemic lupus erythematosus (SLE) is often considered a prototypic autoimmune disease. The SLEDAI (an index of disease activity in SLE) and SLICC (a damage index) were positively associated with PCSK9 levels [83]. The results showed that most of the lipid molecules including LDL-C, LDL: High density lipoprotein (HDL) ratio, non-HDL, apolipoprotein (Apo) A1, Apo B and PCSK9 levels were lower in SLE patients than in controls, with the exception of HDL cholesterol. Another clinical study identified a role for PCSK9 in SLE and suggested that PCSK9 may exert an adverse effect on SLE activity and complications caused by SLE. Researchers suggested that one of the underlying reasons for the association of PCSK9 with disease activity may be that oxLDL promoted the activation of DCs, which was dependent on PCSK9 expression. Silencing of PCSK9 eliminated this effect [84]. A paper published in 2020 by Hiurma et al. reported that most lipid-related molecules were decreased in patients with SLE compared with controls and this downregulation included PCSK9. However, SLE patients with higher disease activity and damage exhibited higher PCSK9 serum levels [71]. Therefore, we might draw a preliminary conclusion that PCSK9 impacts disease activation is dependent on the plasma cholesterol levels. In the future, we anticipate more studies to draw a more definite conclusion about their correlations.

Antiphospholipid antibodies (aPLAs) are commonly observed in patients with SLE and can also manifest as miscarriage, an important risk factor for CVD. PCSK9 seems to be associated with aPLAs. The LDLR gene was first shown to be associated with thrombosis in patients with aPLAs. In addition, a significant correlation was also observed with the PCSK9 gene in this patient group [85]. PCSK9 might also play a role in other autoimmune diseases, such as rheumatoid arthritis (RA) [86]. In psoriasis, PCSK9 was also shown to be an adverse factor. One study showed that 3 months of methotrexate monotherapy reduced PCSK9 levels and improved psoriasis, indicating that PSCK9 could be used as a marker of psoriasis activity [87]. In the development of these autoimmune diseases, such as SLE, RA, and psoriasis, PCSK9 levels are strongly correlated with disease activity and severity. An exploration of the cellular mechanisms involved in PCSK9 and immune regulation has revealed that T cells play an important role. In particular, DCs deliver antigens to T cells, which further influences the T-cell response.

Overall, PCSK9 is not only involved in infectious disease processes but is also closely related to noninfectious inflammation, including lipid metabolism and autoimmune diseases. It can not only be used as a therapeutic target but can also be used as a prognostic predictor of the degree of inflammation or activation. PCSK9 is involved in inflammation through its interaction with LOX-1, and LDLR enhances lipid scavenging by pathogens and regulates the inflammatory state with the help of sirtuins and some proinflammatory cytokines. PCSK9 can even be used in the treatment and prognostic evaluation of inflammation before the exact mechanism has been clarified.

## PCSK9 and different subtypes of T cells

### PCSK9 and CD8 + T cells

Before T-cell activation after stimulation, antigen-presenting cells (APCs) promote antigen binding to T-cell receptors (TCRs). The CD8 molecule is a leukocyte differentiation antigen that is a glycoprotein expressed on the surface of some T cells and is used to assist TCRs in recognizing antigens and participating in the transduction of T-cell activation signals, also known as TCR coreceptors. The CD8 + T cells typically differentiate into CTLs upon activation and specifically kill target cells. The CTL response is an important defense against viral invasion and is also a key effector of tumor immunity [88, 89]. CD8 + T cells are an important branch of adaptive immunity that helps to clear intracellular pathogens and conduct an effective antitumor immune response [90]. In the activated state, T cells begin to proliferate, differentiate, and release cytokines to coordinate immune responses, including promoting cytotoxicity and enhancing B-cell antibody production [91, 92]. In recent years, some evidence has indicated that excessive CD8 + T-cell function leads to self-tissue damage and the development of autoimmune diseases such as type 1 diabetes (T1D), Crohn’s disease, multiple sclerosis (MS), and autoimmune arthritis [93–96].

**Fig. 3:**
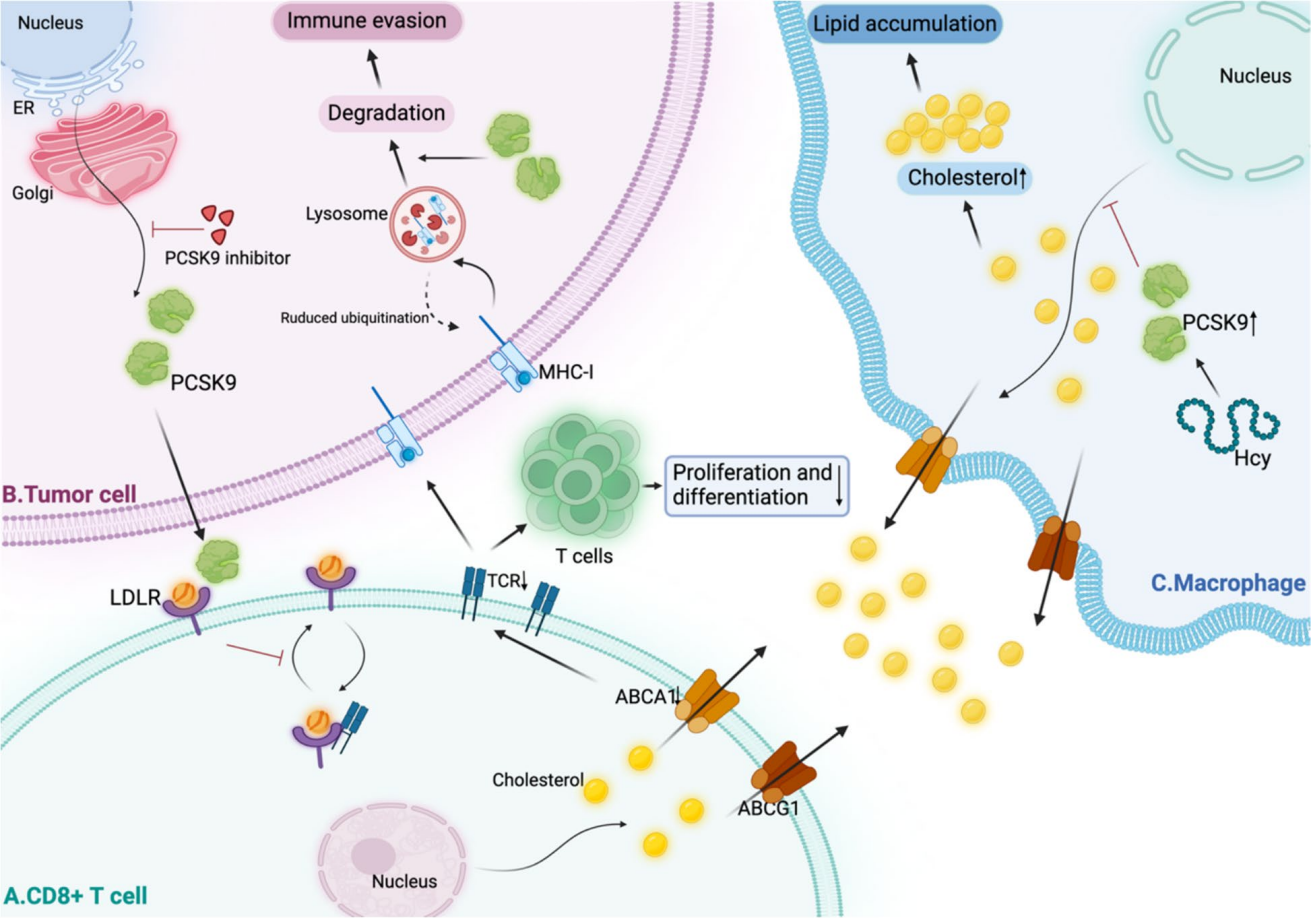
Mechanisms underlying different regulatory roles of PCSK9 in CD8 + T cells, tumor cells, and macrophages (created in https://BioRender.com). **A** ABCA1 and ABCG1 can regulate intracellular cholesterol efflux. The binding of PCSK9 to LDLR prevents the recirculation of LDLR and TCRs to the plasma membrane, thereby inhibiting the effector function of cytotoxic T cells. **B** Description of the production of PCSK9. PCSK9 physically binds to MHC-I to promote lysosomal degradation in tumor cells, decreasing the presentation of MHC-I on the cell surface and promoting immune escape from the tumor. **C** Hcy-induced PCSK9 inhibits cholesterol efflux to the extracellular compartment via ABCA1 and ABCG1, which results in an increase in intracellular cholesterol levels and ultimately lipid accumulation in macrophages. The representation in Fig. 3 is the preliminary speculation and the definite situation requires further confirmation in the future

During lipid metabolism, LDL enters T cells, and LDLR interacts with the TCR complex to regulate TCR recycling and signaling, leading to CD8 + T-cell proliferation and activation, which results in increased CTL numbers [97, 98]. PCSK9 can downregulate LDLR levels and TCR signals in CD8 + T cells, preventing the recirculation of LDLR and TCRs to the plasma membrane and thereby inhibiting the effector function of CTLs. In addition, the inhibition of PCSK9 in tumor cells can enhance the antitumor activity of CD8 + T cells by attenuating the inhibitory effect on CD8 + T cells, thus inhibiting tumor progression [98]. Previous reports have shown that cholesterol on cell membranes functions in the recycling of major histocompatibility complex class I (MHC-I) molecules [99]. An article published in *Nature* by Liu et al. confirmed inhibition of PCSK9 through genetic deletion or using PCSK9 antibody promoted massive infiltration of CD8 + cytotoxic T cells within tumors in 2020. They indicated the potential mechanisms might be that PCSK9 promoted lysosomal degradation by physically binding to MHC-I, disrupting the recirculation of MHC-I to the tumor cell surface and thus reducing cell surface MHC-I signaling [55] (Fig. 3).

Adorni et al. reported that PCSK9 could directly inhibit the expression of the ATP-binding cassette subfamily A 1 (ABCA1) mRNA and protein in macrophages, thereby inhibiting cholesterol efflux [100]. ABCA1 not only is closely related to cholesterol homeostasis but also regulates TCR signaling in CD4-positive (CD4 +) and CD8 + T cells. Lack of ABCA1 in T cells can lead to impaired TCR signaling, inhibiting T-cell survival, proliferation, and differentiation [101]. ATP-binding cassette subfamily G 1 (ABCG1) is highly expressed in a variety of immune cells, including macrophages and T lymphocytes, and plays a role in the intracellular transport of cholesterol. Bensinger et al. described the importance of ABCG1 in the proliferative activation of T cells through mechanisms linking cellular lipid metabolism and proliferation [102]. Jin P et al. reported that SBC-115076 (a PCSK9 inhibitor) could reverse the homocysteine (Hcy)-induced downregulation of ABCA1 and ABCG1, lipid accumulation, and inhibition of cholesterol efflux. In other words, PCSK9 might promote lipid accumulation by inhibiting macrophage ABCA1- and ABCG1-mediated cholesterol efflux [103] (Fig. 3). In addition, invariant NKT (iNKT) cells, which recognize glycolipid antigens, are a subpopulation of T lymphocytes. The results reported by Sag D et al. suggested that alterations in intracellular cholesterol homeostasis induced by ABCG1 profoundly affect the development and function of iNKT cells [104].

Other studies have indicated a possible association between PCSK9 and T-cell function via several mediators. Sterol-regulatory element binding proteins (SREBPs) are a family of transcription factors involved in the biosynthesis of cholesterol, fatty acids, and triglycerides that control the expression of genes important for lipid synthesis and uptake. In recent years, SREBPs were found to be upregulated in a variety of tumors, to promote tumor growth, and to provide a mechanistic link between altered lipid metabolism and malignancy [105–107]. The PCSK9 promoter contains a sterol regulatory element, indicating that PCSK9 transcription is dependent on sterol [108]. Three isoforms of SREBP, SREBP1a, SREBP1c, and SREBP2, have been identified, each of which plays a different role in lipid synthesis. SREBP2 mainly transactivates genes encoding molecules involved in cholesterol biosynthesis, intracellular lipid movement, and lipoprotein import [109]. SREBP2 had been shown to upregulate PCSK9 expression in several previous studies [110–112]. In addition to their functions in lipid metabolism, Kidani Y et al. showed that SREBPs are essential for the metabolic reprogramming of CD8 + T cells and can affect the growth, proliferation, and survival of CD8 + T cells. In the absence of SREBP signaling, CD8 + T-cell proliferation is reduced, and the antiviral immune response is subsequently diminished [113].

### PCSK9 and CD4 + T cells

CD4 + T cells, along with CD8 + T cells, comprise the majority of mature T lymphocytes [114]. CD4 + T cells are important mediators of innate and antigen-specific immune responses, assisting CD8 + T cells and B lymphocytes in facilitating pathogen clearance [115]. CD4 + T cells can be activated and differentiated into different Th cell subsets, namely, Th1, Th2, Th17, and Treg cells, each of which induces different effector functions in response to different antigenic stimuli and cytokine signals [116]. Treg cells represent 5–10% of the peripheral blood (PB) CD4 + T-cell population and play a central role in modulating various immune responses and silencing self-reactive T cells [117].

We mentioned above that ABCA1 regulates TCR signaling in CD4 + T cells, thereby affecting T-cell proliferation and differentiation. With respect to ABCG1, despite its function in the process of T-cell proliferation and activation, Wojcik et al. also confirmed that it might play an important role in the homeostatic function of T cells [118]. ABCG1 can regulate the ability of T cells to differentiate into Tregs. When ABCG1 was selectively deleted in the Tregs of LDL receptor-deficient (LDLR-deficient) mice, a 30% increase in the Treg percentage was observed in the mouse aorta and aortic draining lymph nodes (LNs). Moreover, increased differentiation of naive CD4 + T cells to Tregs was observed in the absence of ABCG1 [119]. By examining the relationship between serum PCSK9 levels and disease activity in patients with ankylosing spondylitis (AS), Cai et al. reported that the serum PCSK9 level was positively correlated with disease activity and the proportion of Th17 cells among CD4 + T cells [120]. In another study, they reported that PCSK9 promoted Th1 and Th17 cell differentiation in individuals with AS [117]. A similar effect was observed on RA patients, where PCSK9 was positively correlated with the proportion of Th17 cells and the increase in the Th17/Treg ratio [121].

The exact mechanism by which PCSK9 interacts with T cells is unclear, and ABCA1, ABCG1, and SREBPs, especially SREBP2, might be promising targets for exploring the function of CD8 + T cell underregulation by PCSK9. In addition, PCSK9 was found to upregulate the number of Th1 and Th17 cells and promote differentiation in patients with autoimmune diseases. More and larger sample size studies are needed to deeply explore mechanisms between them in the future.

## Signaling pathways associated with PCSK9 during the immune response

### Mitogen-activated protein kinase (MAPK) signaling pathway

The MAPK signaling pathway is one of the most important signaling pathways in the eukaryotic signaling network, and its subfamilies mainly include extracellular-signal-regulated kinase (ERK), p38, and C-jun NH_2_-terminal kinase (JNK). The MAPK signaling pathway regulates a wide range of physiological and cancer-related cellular processes and is primarily involved in a wide range of cellular functions, including cell proliferation, differentiation, and apoptosis [122].

T-cell senescence can be regulated by MAPK signaling. MAPK signaling inhibitors such as U0126 and SB203580 can prevent lung, breast, and melanoma tumor cell- and Treg cell-mediated T-cell senescence, thereby enhancing antitumor immunity and the efficacy of immunotherapy in vivo [123]. Interestingly, researchers proposed that MAPK signaling inhibitors combined with an anti-programmed death-ligand 1 (anti-PD-L1) antibody exerted a synergistic effect. A similar result was confirmed by Sengal A. and his colleagues, who observed a significantly reduced number of CD8 + T cells, highlighting the combination of MAPK and ICIs as a potential immunotherapeutic strategy [124].

PCSK9 could regulate the MAPK signaling pathway at the molecular level through a specific protein (heat shock protein 70, HSP70), a member of the evolutionarily highly conserved heat shock protein family, or by directly affecting p38 MAPK and JNK. PCSK9 has been reported to play an important role in the genesis, development, and prognosis of GC by promoting the MAPK signaling pathway through upregulation of HSP70, thereby promoting GC metastasis and inhibiting apoptosis [62]. Downregulation of PCSK9 by short hairpin RNA (shRNA)-PCSK9 affected the expression of apoptosis related proteins and MAPK pathway proteins induced by ox-LDL. Li J et al. reported that shRNA-PCSK9 significantly reduced the phosphorylation of p38 and JNK, thereby promoting endothelial cell apoptosis through the JNK/p38 MAPK pathway [125]. The p38 MAPK regulates T-cell activation by selectively activating nuclear factor of activated T cells c1 (NFATc1). NFATc1 can control the cytotoxicity and metabolic switching of activated CD8 + T cells [126]. Wu et al. showed that NFATc is the direct target of p38 MAPK and that its overall effect is significant [127]. JNKs can regulate human physiological processes, including embryonic development, immune effects, and neuronal function, by modulating gene expression, cell death/survival pathways, and cytoskeletal protein dynamics. Conze D et al. reported that the deletion of JNK1 enhanced IL-2 production and the proliferation of CD8 + T cells, but this effect was not observed on CD4 + T cells [128].

In addition, PCSK9 has been shown to promote ox-LDL-induced endothelial cell apoptosis through the Bcl-2/Bax-Caspase 9/3 signaling pathway. Their research experiments suggested that PCSK9 might activate the Bcl-2/Bax-Caspase 9/3 pathway by affecting apoptosis related proteins such as caspase 3, caspase 9, Bcl-2, Bax [125, 129]. PCSK9 plays an important role in the proliferation of CD8 + T cells through the MAPK signaling pathway and is involved in the apoptosis of endothelial cells. However, the exact mechanism by which PCSK9 affects T-cell subtypes should be investigated in additional studies (Fig. 4①).

**Fig. 4:**
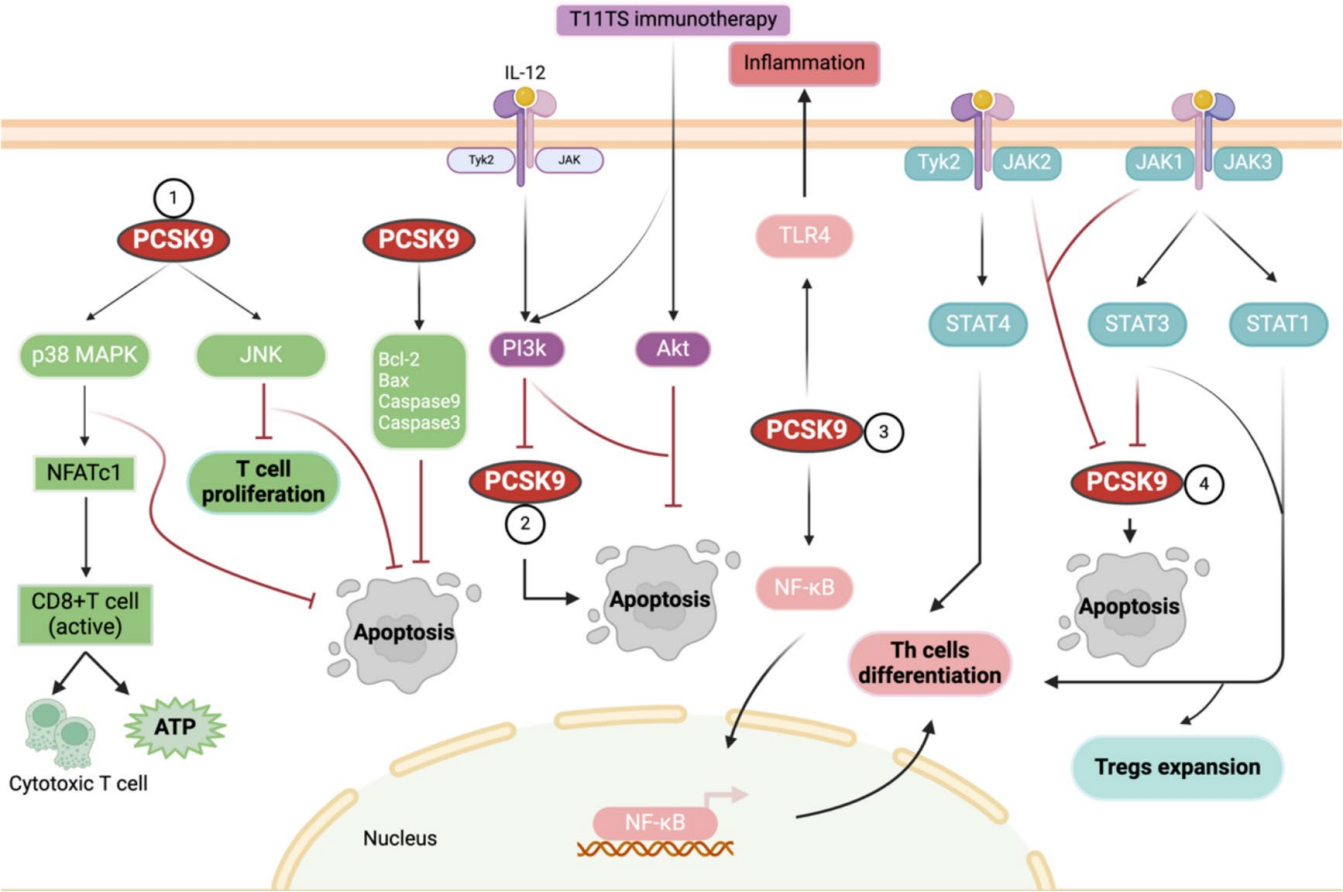
Signaling pathways associated with PCSK9 during the immune response (created in https://BioRender.com). ① MAPK signaling pathway. MAPK regulates cytotoxicity and metabolic switching in activated CD8 + T cells through the activation of NFATc1. PCSK9 is involved in endothelial cell apoptosis through the MAPK and Bcl-2/Bax-Caspase 9/3 signaling pathways. ② PI3K/Akt signaling pathway. IL-12 and T11TS immunotherapy can protect T cells from apoptosis and promote their survival through the PI3K/Akt pathway. Inhibition of PCSK9 by PI3K/Akt promotes apoptosis. ③ NF-κB signaling pathway. PCSK9 promotes Th cells differentiation through the activation of the NF-κB signaling pathway. PCSK9 promotes vascular inflammatory responses through the upregulation or activation of the TLR-4/NF-κB pathway. ④ JAK/STAT signaling pathway. JAK/STAT promotes the differentiation of Th cells and the expansion of Tregs. PCSK9 expression in different cells is affected by JAK/STAT-related cytokines or kinases

### The phosphatidylinositol 3-kinase (PI3K)/protein kinase B (Akt) signaling pathway

The PI3K/Akt signaling pathway is an intracellular signaling pathway involved in many biological processes, including cell metabolism, growth, survival, proliferation, and apoptosis. These processes are mediated by serine or threonine phosphorylation of a series of downstream substrates, and the crucial genes involved are PI3K and Akt/protein kinase B [130, 131].

Apremilast (a phosphodiesterase-4 inhibitor) was found to regulate Th17 and Treg cells by inhibiting the phosphorylation of the PI3K/Akt/Forkhead Box O1 (FoxO1) signaling pathway, thereby alleviating experimental autoimmune uveitis (EAU) [132]. The role of E proteins (transcriptional activators or repressors with the ability to bind specific DNA sequences termed E-box sites) during the differentiation of mature CD4 + T cells has been emphasized, especially in the maintenance of Treg and Th17 cells [133]. In turn, E protein activity can be a new player involved in the fine regulation of resting CD4 + T-cell activity and activation, partly due to the regulation of TCR signaling by the PI3K/Akt pathway [134].

Juntilla MM et al. discovered that the PI3K/Akt signaling pathway plays an important role in T-cell development. Some critical signals, such as those from pre-TCR, Notch, and IL-7 receptors (IL-7Rs), are known to determine the differentiation of cells from the CD4-CD8- (double-negative) phase to the CD4 + CD8 + (double-positive) phase [135]. The PI3K/Akt signaling pathway transforms these extracellular signaling events into multiple endpoints, including cell proliferation, differentiation, survival, and allelic exclusion.

Kawabe K et al. reported that the mechanism by which IL-12 protects T cells from glucocorticoid-induced apoptosis involves the PI3K/Akt pathway and the induction of glucocorticoid modulatory element binding protein (GMEB1) [136]. In addition, the results from a glioma-loaded animal experiment indicated that the PI3K/Akt pathway was important for the survival of activated downstream T cells. A previous study documented that immunotherapy with the new immunotherapeutic molecule T11-targeted structure (T11TS) could improve T-cell survival by activating the PI3K/Akt pathway in T cells [137].

It has been reported that SREBP2 could upregulate PCSK9 expression [138], and PI3K/Akt was known to regulate SREBP expression [139]. Blockade of SREBP signaling leads to dysregulated activation of PI3K in intratumoral Treg cells, which results in impaired function of Treg cells [140]. Wu et al. indicated that PCSK9 expression in HCCs was significantly downregulated by the activation of the PI3K/Akt-SREBP2 signaling pathway [141]. In their exploration of the underlying molecular mechanisms, data showed the involvement of PI3K/Akt phosphorylation. The PI3K/Akt inhibitor LY294002 abolished the effects of reduced PCSK9 and SREBP2 expression, suggesting a PI3K/Akt-mediated mechanism. The PI3K/Akt pathway had also been found to be involved in hydrogen sulfide (H2S)-mediated regulation of PCSK9 expression. The potential mechanisms revealed that the level of Akt phosphorylation was increased and the expression of SREBP-2 and PCSK9 was correspondingly decreased [142].

As mentioned above, certain agents, such as phosphodiesterase-4 inhibitors, can modulate Th17 and Treg cells through the PI3K/Akt signaling pathway. Notably, E proteins are not only useful in promoting the maintenance of Th17 cells and Tregs during CD4 + T-cell differentiation but also in regulating CD4 + T-cell quiescence and activation via the PI3K/Akt signaling pathway. T11TS immunotherapy and IL-12 protect T cells from apoptosis and promote their survival through the PI3K/Akt signaling pathway, respectively. Overall, the PI3K/Akt signaling pathway plays a significant role in promoting T-cell proliferation, differentiation, and survival. The expression of PCSK9 in different cells can be regulated by the PI3K/Akt signaling pathway by mechanisms mainly related to the direct effect of PI3K and Akt phosphorylation (Fig. 4②).

### The Toll-like receptor-4 (TLR-4)/nuclear factor kappa-B (NF-κB) signaling pathway

Toll-like receptors (TLRs) are pattern recognition receptors in the innate immune system. They can activate downstream inflammatory signaling pathways to combat the invasion of exogenous microbial pathogens [143, 144]. Activation of the TLR signaling pathway is generally accompanied by the activation of NF-κB, which in turn facilitates the release of proinflammatory cytokines that trigger leukocyte aggregation and inflammation [145].

NF-κB acts as a downstream effector of the TLR4 signaling pathway and mediates a variety of inflammatory processes [146]. The NF-κB family of transcription factors is crucial for the development, maintenance, and function of Tregs [147]. The development of different T-cell subsets is dependent on several known transcription factors, including forkhead box protein 3 (FoxP3) for Tregs and retinoid-related orphan receptor (ROR) γt and RORα for Th17 cells [148]. More than a decade ago, Ruan et al. identified a possible role for NF-κB in regulating RORγt gene expression in Th17 cell differentiation and function [149]. Hovelmeyer N et al. revealed a regulatory role of NF-κB transcription factors in the development and functions of Tregs in genetic mouse models [150]. The molecular recognition of the NF-κB pathway in Treg cells holds promise for novel immunotherapeutic strategies for autoimmune or cancer therapies.

A study was conducted to explore whether PCSK9 could play a role in macrophage inflammation through the TLR4/NF-κB signaling pathway. After lipid loading, TLR4 protein expression was reduced in PCSK9-silenced inflammatory cells, and NF-κB nuclear translocation was decreased, suggesting the downregulation of the proinflammatory TLR4/NF-κB pathway. In turn, PCSK9 upregulated the TLR4/NF-κB signaling pathway and promoted inflammatory responses [151]. Huang et al. reported that PCSK9 expression is significantly increased during the occurrence of sepsis and can induce an inflammatory response through the activation of the TLR4/MyD88/NF-κB and NLRP3 pathways, which can lead to vascular endothelial dysfunction [152]. Their data confirmed that inhibition of PCSK9 prevented TLR4 activation during sepsis, improved vascular function and increased survival in septic mice.

Evolocumab downregulated TLR4 levels in rat retinal Müller cells (RMCs) and exerted anti-inflammatory effects by negatively regulating the activation of the TLR4 signaling pathway [153]. Tang et al. explored the role of PCSK9 in vascular inflammation that promotes the progression of atherosclerosis and reported that PCSK9 gene disruption directly inhibited atherosclerosis by reducing vascular inflammation and inhibiting the TLR4/NF-κB signaling pathway without affecting plasma cholesterol levels in ApoE KO mice fed a high-fat diet [154]. These experimental results above demonstrated that PCSK9 could regulate the TLR4/NF-κB signaling pathway through TLR4 independent of cellular lipid metabolism.

PCSK9 can promote the differentiation of naive CD4 + T cells into Th1 and Th17 cells and upregulate the secretion of cytokines to induce vascular inflammation [155]. Cai J et al. reported that PCSK9 promoted Th cell differentiation in AS, suggesting that PCSK9 promotes Th1 and Th17 cell differentiation through the activation of the NF-κB pathway [156].

To summarize, PCSK9 promotes Th1 and Th17 differentiation through the activation of the NF-κB signaling pathway. PCSK9 promotes vascular inflammatory responses by upregulating or activating the NF-κB signaling pathway. In contrast, the inhibition of PCSK9 suppresses the NF-κB signaling pathway and thus inhibits inflammatory responses (Fig. 4③).

### The Janus kinase (JAK)/signal transducer and activation of transcription (STAT) signaling pathway

The JAK/STAT pathway constitutes the principal signaling cascade for a large number of cytokines and growth factors [157]. Dysregulation of the JAK/STAT signaling pathway can lead to autoimmunity, allergic diseases, and cancer [158]. The JAK/STAT pathway has been well studied in terms of its regulation of T-cell differentiation [159]. Moreover, different STAT molecules, such as STAT1, STAT3, and STAT4, are required for both CD8 + cytotoxic T cell developmental processes and transcriptional regulation [158].

Oncostatin M (OSM) is a member of the IL-6 family of cytokines that are produced primarily by activated T cells and macrophages [160]. OSM induces fibroblast proliferation, inhibits tumor cell proliferation, and plays a role in immune regulation [161]. A study by Cao et al. showed that OSM suppressed hepatic PCSK9 mRNA and protein expression in hypercholesterolemic hamsters in vivo. The expression of PCSK9 dependented on the kinase pathways of JAK1/2 and MEK1 (mitogen-activated protein kinase kinase 1)/ERK. This finding revealed a cytokine-triggered regulatory network of PCSK9 expression associated with the JAK and ERK signaling pathways [162]. This regulatory network might facilitate further exploration of disease treatment strategies in combination with statins. Wicinski M et al. reported that PCSK9 can be altered by tumor-associated pathways that utilize kinases such as PI3K/Akt, JNK, and JAK/STAT. PCSK9 is an important effector involved in the activity of the JNK pathway, PI3K and ERK1/2, by affecting the degradation of the ApoE 2 receptor [163]. Ruscic et al. described the effect of TNF-α on the JAK/STAT pathway and found that the negative regulator of this pathway is suppressor of cytokine signaling 3 (SOCS3). The increased expression of SOCS3 led to the inhibition of STAT3 phosphorylation, which in turn increased PCSK9 expression in hepatocytes [164].

In summary, STAT molecules promote Th cell differentiation and Treg expansion, while STAT3 phosphorylation and JAK1/2 inhibit PCSK9 expression. PCSK9 expression in different cells is susceptible to JAK/STAT-related cytokines or kinases (Fig. 4④). Agents targeting the JAK/STAT pathway, such as ruxolitinib, have been confirmed to improve graft-versus-host disease (GVHD) or severe ‘cytokine storms’ in some serious conditions. From the above studies, we conclude that PCSK9 regulates different signaling pathways by direct action on different kinases in a molecular mechanism that is not dependent on cellular lipid metabolism. We expect more and more in-depth studies to explore the molecular mechanisms by which PCSK9 regulates different signaling pathways.

## Conclusions

In this review, we summarized the biological processes involved, the relationships of different subtypes of T cells with PCSK9, and the signaling pathways associated with PCSK9. In addition to its important role in lipid metabolism, PCSK9 is involved in T-cell differentiation, activation, subsequent immune responses, and even tumorigenesis. In the future, targeting PCSK9 might be a promising strategy for exploring the modulatory function of PCSK9 in the T-cell response and in some abnormal immune activities, such as autoimmune diseases or GVHD posttransplantation.

## Data Availability

No datasets were generated or analysed during the current study.

## Abbreviations

PCSK9: Proprotein convertase subtilisin/kexin type 9
NARC-1: Neuroapoptosis-regulated converting enzyme-1
PC: Proprotein convertase
LDLR: Low-density lipoprotein receptor
OxLDL: Oxidized low-density lipoprotein
LDL: Low-density lipoprotein
CTLs: Cytotoxic T lymphocytes
Th cells: Helper T cells
Tregs: Regulatory T cells
3D: Three-dimensional
bp: Base pairs
CDS: Coding sequence
aa: Amino acids
SP: Signal peptide
CHRD: C-terminal Cys/His-rich-domain
ER: Endoplasmic reticulum
GOF: Gain of function mutation
LOF: Loss of function mutation
mAbs: Monoclonal antibodies
EGF-A: Epidermal growth factor-like repeat A
LDL-C: Low-density lipoprotein cholesterol
CAP1: Cyclase-associated protein 1
FH: Familial hypercholesterolemia
CHD: Coronary atherosclerotic heart disease
siRNA: Small interfering RNA
VLP: Virus-like particle
GC: Gastric cancer
PD-1: Programmed cell death-1
ICIs: Immune checkpoint inhibitors
CRC: Colorectal cancer
HCC: Hepatocellular carcinoma
NSCLC: Non-small cell lung cancer
IHC: Immunohistochemistry
mPFS: Median progression-free survival
ORR: Objective response rate
DCR: Disease control rate
ERS: Endoplasmic reticulum stress
SRs: Scavenger receptors
LOX-1: Oxidized LDL receptor-1
DCs: Dendritic cells
GO: Graves orbitopathy
SLE: Systemic lupus erythematosus
HDL: High density lipoprotein
Apo: Apolipoprotein
aPLAs: Antiphospholipid antibodies
RA: Rheumatoid arthritis
APCs: Antigen-presenting cells
TCRs: T-cell receptors
CD8 + T cells: CD8-positive T cells
T1D: Type 1 diabetes
MS: Multiple sclerosis
MHC-I: Major histocompatibility complex class I
ABCA1: ATP-binding cassette subfamily A 1
CD4 + T cells: CD4-positive T cells
ABCG1: ATP-binding cassette subfamily G 1
Hcy: Homocysteine
iNKT: Invariant NKT
SREBPs: Sterol-regulatory element binding proteins
PB: Peripheral blood
LNs: Lymph nodes
AS: Ankylosing spondylitis
MAPK: Mitogen-activated protein kinase
ERK: Signal-regulated kinase
JNK: C-jun NH_2_-terminal kinase
anti-PD-L1: Anti-programmed death-ligand 1
HSP70: Heat shock protein 70
shRNA: Short hairpin RNA
NFATc1: Nuclear factor of activated T cells c1
PI3K: Phosphatidylinositol-3-kinase
Akt: Protein kinase B
FoxO1: Forkhead Box O1
EAU: Experimental autoimmune uveitis
IL-7Rs: IL-7 receptors
GMEB1: Glucocorticoid modulatory element binding protein
T11TS: T11-targeted structure
H2S: Hydrogen sulfide
TLR-4: Toll-like receptor-4
NF-κB: Nuclear factor kappa-B
TLRs: Toll-like receptors
FoxP3: Forkhead box protein 3
ROR: Retinoid-related orphan receptor
RMCs: Retinal Müller cells
JAK: Janus kinase
STAT: Signal transducer and activation of transcription
OSM: Oncostatin M
MEK1: mitogen-activated protein kinase kinase 1
TNF-α: Tumor necrosis factor-α
SOCS3: Suppressor of cytokine signaling 3
GVHD: Graft-versus-host disease
ASCVD: Atherosclerotic cardiovascular disease
AMI: Acute myocardial infarction
L – IFPTA: Liposomal Immunogenic Fused PCSK9-Tetanus peptide plus Alum adjuvant
CETP: Cholesterol ester transfer protein
